# Herbgenomics meets Papaveraceae: a promising -omics perspective on medicinal plant research

**DOI:** 10.1093/bfgp/elad050

**Published:** 2023-11-10

**Authors:** Natalia Kielich, Oliwia Mazur, Oskar Musidlak, Joanna Gracz-Bernaciak, Robert Nawrot

**Affiliations:** Department of Molecular Virology, Institute of Experimental Biology, Adam Mickiewicz University, Poznań, Poland; Department of Molecular Virology, Institute of Experimental Biology, Adam Mickiewicz University, Poznań, Poland; Department of Molecular Virology, Institute of Experimental Biology, Adam Mickiewicz University, Poznań, Poland; Department of Molecular Virology, Institute of Experimental Biology, Adam Mickiewicz University, Poznań, Poland; Department of Molecular Virology, Institute of Experimental Biology, Adam Mickiewicz University, Poznań, Poland

**Keywords:** herbal medicine, herbgenomics, medicinal plants, -omics, Papaveraceae

## Abstract

Herbal medicines were widely used in ancient and modern societies as remedies for human ailments. Notably, the Papaveraceae family includes well-known species, such as *Papaver somniferum* and *Chelidonium majus*, which possess medicinal properties due to their latex content. Latex-bearing plants are a rich source of diverse bioactive compounds, with applications ranging from narcotics to analgesics and relaxants. With the advent of high-throughput technologies and advancements in sequencing tools, an opportunity exists to bridge the knowledge gap between the genetic information of herbs and the regulatory networks underlying their medicinal activities. This emerging discipline, known as herbgenomics, combines genomic information with other -omics studies to unravel the genetic foundations, including essential gene functions and secondary metabolite biosynthesis pathways. Furthermore, exploring the genomes of various medicinal plants enables the utilization of modern genetic manipulation techniques, such as Clustered Regularly-Interspaced Short Palindromic Repeats (CRISPR/Cas9) or RNA interference. This technological revolution has facilitated systematic studies of model herbs, targeted breeding of medicinal plants, the establishment of gene banks and the adoption of synthetic biology approaches. In this article, we provide a comprehensive overview of the recent advances in genomic, transcriptomic, proteomic and metabolomic research on species within the Papaveraceae family. Additionally, it briefly explores the potential applications and key opportunities offered by the -omics perspective in the pharmaceutical industry and the agrobiotechnology field.

## Teaser

Short Abstract/Highlights

Herbgenomics integrates all high-throughput data, providing a deeper understanding of the genetic background behind the medicinal properties of bioactive molecules within the Papaveraceae family. This knowledge can be leveraged in the discovery of plant-based drugs.

## INTRODUCTION

Plants produce a vast diversity of specialized metabolites that allow them to interact with their environment and respond to various abiotic and biotic stresses. Some of these molecules have the potential to be exploited as pharmaceuticals. Since ancient times, herbs and different parts of plants, such as seeds, roots, leaves and fruits, as well as their extracts, have been applied to treat a broad range of diseases. Initially, their usage was instinctive and based on the experiences of the local population [1]. Over the years, with progress in science, the beneficial role of plants in healthcare and their ethnopharmacological attributes were investigated and well described. Nowadays, a variety of natural products, such as aspirin, morphine, noscapine, rivastigmine, oseltamivir, taxol and veregen, are clinically applied as healing agents [2–5]. According to the PubMed database, over the last 5 years, more than 9000 studies related to ‘medicinal plants’ and ‘ethnopharmacology’ have been published. These data have paved the way for further investigation of herbal medicinal products. Recently, both research facilities and drug companies have shown increased attention toward natural plant products as potential targets for discovery and the development of new bioactive molecules [6]. The driving forces behind this new interest include the growing human population, numerous unsuccessful clinical trials and therapies, unwanted side effects of synthetic drugs and the rise of antimicrobial resistance [7]. Currently, the usage of plant medicines is regarded as an alternative to conventional medicine, with plant extracts commonly employed as part of traditional therapies [8]. To transform herbal materials into botanical drugs, several factors must be considered, including the identification of the plant species, selection, extraction and purification of active molecules, *in vivo* and *in vitro* bioassays, quality control, nonclinical safety assessment and clinical efficacy [9–11]. Consequently, plant-derived natural therapy requires documented scientific evidence with statistical significance, leading not only to the discovery of new medicinal compounds but also to a deeper and more comprehensive understanding of plant biology, physiology and taxonomy [12].

**Figure 1:**
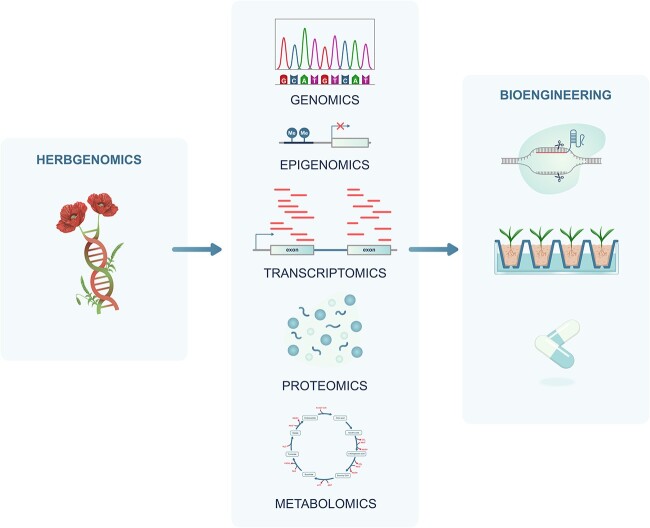
Herbgenomics is a field of research that combines high-throughput -omics tools to understand the molecular mechanisms underlying the medicinal properties of herbs, which could be applied in both genetic bioengineering and medicinal plant breeding.

Following the revolution in biology, which saw the sequencing of the first plant genome of *Arabidopsis thaliana* in 2000, high-throughput sequencing technology provided an important breakthrough in fundamental herbal research and its potential applications [13, 14]. Genomics, along with its related studies, plays a crucial role in identifying genes involved in plant-specialized metabolism. Consequently, traditional herbal medicine has entered a new era known as ‘herbgenomics’ [15, 16] or ‘herbal genomics’ [17]. This new discipline integrates various -omics fields, such as genomics, transcriptomics, epigenomics, proteomics, metabolomics and bioinformatics, offering powerful tools for understanding and controlling the genetic and biological aspects of herbs (Figure 1). It involves next-generation sequencing (NGS), protein purification as well as identification and even genome editing [18]. Most importantly, the new approach of herbgenomics explores the molecular genetics of herbs at both the single-cell and individual levels. Furthermore, it helps to merge all -omics data to enhance the genetic foundations responsible for the biological activity of medicinal plants. Systematically, herbgenomics unravels the biosynthesis and regulatory pathways of secondary metabolites, which have the potential to serve as pharmaceutical compounds. Herbgenomics broadens the opportunities for improving the breeding of medicinal plants and enhancing their metabolic bioengineering. Additionally, it offers a comprehensive method for constructing herbal gene libraries that contain identified and investigated biomolecules. Undoubtedly, herbgenomics sheds new light on folk medicine from an -omics perspective.

In this review, we would like to focus on the Papaveraceae family, which serves as an illustrative example of a plant family with a rich history of medicinal use. The growing interest in this plant family as a potential pharmaceutical resource has led to the discovery of numerous secondary metabolites in diverse species of these herbaceous plants. This article provides a summary of research conducted in the fields of genetics, transcriptomics, proteomics and metabolomics. Apart from that, we shed light on the practical applications of herbgenomics and how the integration of -omics data can enhance the production of diverse biopharmaceuticals with the medicinal value of herbs belonging to the Papaveraceae family.

## BIOACTIVE COMPOUNDS OF PAPAVERACEAE

Plants belonging to the Papaveraceae family are primarily distributed in the northern temperate to subtropical regions of Europe and Asia. Across this family with 44 genera and 825 species, the genus *Papaver* (poppy) comprises ~50 species [19, 20].

In general, Papaveraceae members are characterized as annual or perennial herbs, shrubs or small trees that produce milky sap, known as latex, in some taxa [21]. The milky sap, produced by laticifers, contains a broad range of biologically active molecules that hold potential medicinal applications. Plant latex extracts have been found to contain numerous proteins and low molecular weight compounds, including alkaloids, carotenoids, phenols and terpenoids, among others [22–26]. Alkaloids, which are nitrogen-containing organic molecules, form the largest group of components within latex and are responsible for the plant’s defense against herbivores and pathogens. Benzylisoquinoline alkaloids (BIAs) represent a diverse group of plant secondary metabolites mostly known for their pharmacological activities, that are widely distributed among various plant families, including the Papaveraceae [27, 28]. In recent years, BIA-derived pharmaceuticals, such as noscapine and berberine, have found utility as anticancer agents, while berbamine and coptisine have been explored as anti-inflammatory drugs [29].

Numerous alkaloids isolated from Papaveraceae herbs have shown promising biological activities and potential for the development of plant-based drugs. For example, *Papaver somniferum* L. has yielded morphine and codeine, which are narcotic analgesics and opioids [30]. These compounds have effects ranging from pain relief and cough suppression to euphoria, sleepiness and addiction due to their impact on the central and peripheral nervous systems [31, 32]. Morphine-based painkillers are widely considered the most effective and inexpensive treatment for severe pain. On the other hand, the dark side of opium poppy is correlated with heroin, a highly addictive narcotic substance introduced to the market by Bayer Pharmaceuticals 200 years ago [33]. Over the years, diacetylmorphine was used as an illicit opioid and had a significant impact on human history in terms of dependence and overdose deaths [34]. Presently, heroin does not have an approved indication for use by the Food and Drug Administration (FDA) [35].

*Chelidonium majus* L., also known as greater celandine, contains compounds, such as isoquinoline alkaloids (IQAs) (e.g. berberine, chelidonine and coptisine), flavonoids, saponins, vitamins and proteins, which contribute to its defense response [24, 36]. In the past, greater celandine was used in European and Traditional Chinese Medicine (TCM) to treat diverse gastric and liver diseases, skin disorders, rheumatoid arthritis, tuberculosis and asthma [37]. Presently, latex extracts from *C. majus* are still used to remove warts, papillae and condylomas caused by human papillomavirus infections [38]. Mostly, the diversity of compounds contained within the latex corresponds to various pharmacological properties, like antibacterial, anti-inflammatory, antiviral, antifungal, hepatoprotective, natriuretic and antidiuretic effects [36, 39]. Both early and recent studies have focused on the composition, antiviral activity and cytotoxic properties of *C. majus* alkaloids [40, 41]. Recent research has indicated a potential therapeutic role of celandine herbs in endometriosis and ovarian cancer [42, 43]. Therefore, celandine’s milky sap holds promise as an anticancer plant-based product, but further detailed analysis is required.

Other poppy species, such as *Eschscholzia californica*, synthesize BIAs like sanguinarine, chelirubine, macarpine and chelerythrine [44]. California poppy, an herb used in traditional medicine, has been employed to induce sleep due to its sedative effects [45, 46]. Many studies have indicated the bioactive properties of *E. californica*, including antibacterial, antifungal, analgesic, anxiolytic and sedative activities [47]. Interestingly, another Papaveraceae species, *Macleaya cordata*, has been commonly used in Asian folk medicine for treating bacterial infections. It yields compounds, such as sanguinarine, protopine and allocryptopine [28]. Pharmaceutical studies have revealed the antimicrobial activity of *M. cordata*, leading to its approval by the European Food Safety Authority (EFSA) as an alternative to antibiotics in food additives for animal production [48].

## DIFFERENT ASPECTS OF HERBGENOMICS IN PAPAVERACEAE

### Genomics

The emergence of plant genomics has provided new insights into the biology and evolutionary history of plant genomes. It has facilitated the identification of genes involved in the biosynthesis of medically important active molecules and has enabled the discovery of new pathways. Furthermore, plant genomics has allowed for the genome-based taxonomic classification of closely related species [49]. Plant genomes typically consist of three genomes: nuclear, chloroplast and mitochondrial. Various events, such as gene duplication, neo-functionalization, conservation of operon-like gene clusters, chromatin modifications and higher-order chromatin structure and dynamics contribute to changes in gene expression [50]. Plant genomes exhibit differences in size, ploidy level, DNA repeats and genome rearrangements, which makes it complicated to identify and understand gene function. The structural complexity of plant genomes enables researchers to decode the molecular and evolutionary processes that have resulted in enormous genomic variation in plants [51–54]. Consequently, annotating plant genomes poses a major challenge for researchers. However, rapid advancements in NGS technology reduced sequencing costs, and the development of bioinformatic analysis tools has greatly facilitated the study of plant genome structures [50]. This has also opened up possibilities for deciphering the genetic makeup of non-model medicinal plants [16]. Despite the complexities associated with plant genomes, six nuclear genomes of Papaveraceae have been successfully sequenced at the chromosomal level. These include *M. cordata*, *E. californica*, *P. somniferum*, *Papaver rhoeas*, *Papaver orientale* and *Corydalis tomentella* [28, 55–58]. The estimated sizes of these genomes are summarized in Table 1.

**Table 1:** Selected available sequenced nuclear and plastid genomes of Papaveraceae species published between 2016 and 2022

Papaveraceae species		Genome size	Sequencing platform	Reference
Common name	Latin name			
Nuclear genome				
–	*Corydalis tomentella*	259 Mb	Illumina HiSeq 4000	[58]
California poppy	*Eschscholzia californica*	502 Mb	Illumina GAII	[55]
Plume poppy	*Macleaya cordata*	378 Mb	HiSeq 2000	[28]
Flanders poppy, Corn poppy	*Papaver rhoeas*	320 Mb	Illumina Novoseq	[65]
Poppy of Troy, Dwarf breadseed poppy	*Papaver setigerum*	589 Mb		
Opium poppy	*Papaver somniferum*	2939 Mb	Illumina, SMRT, PacBio, ONT	[57]
Complete chloroplast genome				
–	*Coreanomecon hylomeconoides* Nakai	158 824 bp	Genome Sequencer FLX System	[83]
–	*Corydalis bungeana Turcz*	167 629 bp	Illumina NovaSeq	[31]
–	*Corydalis edulis*	154 395 bp	Illumina Hiseq2500 platform	[84]
Asian corydalis	*Corydalis fangshanensis*	192 554 bp	Illumina NovaSeq 6000	[85]
–	*Corydalis impatiens*	197 317 bp	Illumina NovaSeq 6000 Sequencing System	[86]
–	*Corydalis inopinata Prain ex Fedde*	181 335 bp	Illumina NovaSeq 6000	[74]
Yanhuanglian	*Corydalis saxicola*	189 029 bp	llumina HiSeq 4000 Platform	[73]
Shaanxi corydalis	*Corydalis shensiana*	155 938 bp	Illumina Hiseq2500 platform	[84]
–	*Corydalis tomentella*	190 247 bp	llumina HiSeq 4000 Platform	[73]
Tsinling Corydalis	*Corydalis trisecta Franch*	161 410 bp	Illumina Hiseq 2000 Platform	[87]
Forest poppy, Japanese poppy	*Hylomecon japonica*	160 011 bp	Illumina HiSeq 2500	[88]
–	*Meconopsis henrici vig.*	153 388 bp	HiSeq2000	[89]
Prickly blue poppy	*Meconopsis horridula Hook. f. & Thomson*	153 761 bp	Illumina HiSeq X	[90]
Lampshade poppy	*Meconopsis integrifolia*	152 714 bp	Illumina Hiseq 2500	[91]
Red poppywort	*Meconopsis punicea*	152 933 bp	HiSeq2000	[92]
Oriental poppy	*Papaver orientale*	152 799 bp	Illumina Hiseq X	[56]
Flanders poppy, Corn poppy	*P. rhoeas*	152 905 bp	Illumina Hiseq X	
Poppy of Troy, Dwarf breadseed poppy	*Papaver setigerum*	152 862 bp	Illumina HiSeq X10	[93]

Due to the close relationship and shared pharmacological activity among many herbs, scientists can determine the differences and similarities between species that belong to the same plant family. It is necessary either to better understand plant evolution or to anticipate the biosynthesis pathways of secondary metabolites for further investigation as therapeutic agents [59–61]. *P. somniferum*, commonly known as opium poppy, is a major medicinal herb and has served as a model species for studying alkaloid metabolism in plants [62]. The results of opium poppy’s BIA production studies over the previous decade revealed the pathways of medicinally important alkaloids, including morphine, noscapine and sanguinarine [27, 28, 63]. Analyzing the opium poppy genome has provided insights into the essential functions of gene duplication and gene clustering in BIA biosynthesis [57]. For example, the fusion of STORR genes, which are involved in the isomerization of (*S*)- to (*R*)-reticuline, a key step in morphine biosynthesis, forms an operon-like gene cluster on chromosome 11. Comparative sequence analysis based on functional genomics, transcriptomics and metabolomics has revealed that the STORR gene fusion is conserved and has persisted since the divergence of the opium poppy from its common ancestor within the *Papaver* genus [64].

The well-established patchwork model, proposed by Ycas (1974) and Jensen (1976), explains how the duplication of genes encoding enzymes, along with variations in substrates, contributes to the construction of metabolic pathways. However, comparative analysis of chromosome-scale plant genomes is still limited in our understanding. In a study by [65], the whole-genome duplications (WGDs) of three *Papaver* genomes were investigated regarding their alkaloid production. The results revealed variations in the biosynthesis of morphine and noscapine among the species, which correlated with post WGD in the ancestor of *P. somniferum* and *Papaver setigerum*. Specifically, *P. somniferum* accumulated the highest amounts of noscapine and morphinan alkaloids. In contrast, *P. rhoeas* synthesized marginal amounts of these alkaloids, while *P. setigerum* produced no noscapine and only trace amounts of morphinan.

With the increasing projects of plant genomes sequencing, an expanding number of biosynthetic gene clusters (BGC), coding enzymes involved in biosynthesis of specialized metabolisms (for instance BIAs), are being discovered [66, 67]. Presently, computational methods like METACLUSTER [68], plantiSMASH [69] or PhytoClust [70] enable the anticipation of BGCs, offering a promising avenue for uncovering novel aspects of plant metabolism and potential new pharmaceutics. Further they can be used in synthetic biology approaches for heterologous metabolites production (see Herbgenomics as an approach in enhanced Papaveraceae biopharmaceutical production) [57].

Chloroplast genomes are increasingly being sequenced and analyzed due to their primarily uniparental inheritance and their vital role in encoding fundamental genes for photosynthesis and plant secondary metabolites [71–73]. Plastid sequences have proven to be powerful tools for resolving evolutionary problems and for identifying species and their relationships across various taxonomic levels [74]. This approach, known as DNA barcode technology, was initially used for microorganisms but is now commonly utilized for accurate species identification across all organisms [75–77].

In recent years, several plastid DNA barcodes have been found and widely applied for the identification of plant species worldwide. Moreover, the chloroplast DNA of several species within the Papaveraceae family has been sequenced (Table 1). The combination of plastid DNA barcodes with -omics data has improved the accuracy of medicinal plant identification compared to traditional methods involving the morphological analysis and chemical identification. Several studies have investigated the complete chloroplast genomes of *Papaver* species. For instance, comparative genome analysis revealed a close relationship between *P. rhoeas*, *P. orientale* and *P. somniferum* [56]. Notable differences were observed in the hypervariable regions, particularly the *ycf1* gene, which is one of the largest genes in these regions and plays a critical role in plant viability. The *ycf1* gene encodes a component of translocon complexes in inner envelope membranes [78]. Due to its variability, the *ycf1* gene is considered a potential barcode for *Papaver* species [56]. Moreover, recent research has reported that *P. somniferum* and *P. setigerum* genomes share considerable homology and have a close phylogenetic relationship despite differences in their ploidy levels. The genome size of *P. somniferum* is ~3.04 Gb, while *P. setigerum* is around 4.9 Gb. Based on genome size and ploidy level, previous reports suggesting that *P. somniferum* is diploid (2*n* = 22) and *P. setigerum* is tetraploid (2*n* = 44) were supported by the authors. Additionally, the authors searched unlinked single nucleotide polymorphism (SNP) between *P. somniferum* accessions, revealing differences in the number of unique alleles among opium poppy samples from ~130 000 SNP sets [79].

Obviously, numerous plant metabolites hold potential pharmacological applications. To achieve this, it is crucial to enhance the molecular identification of medicinal plants. Thus, the whole nuclear and chloroplast plant genomes are urgently required to be sequenced and analyzed. Decades earlier, a series of projects called the ‘Herb Genome Program’ was introduced by Chinese scientists. The purpose of these experiments aimed to sequence and conduct functional analysis on genomes of herbs commonly used in TCM, such as *Ganoderma lucidum* and *Salvia miltiorrhiza* [80, 81]. Another interesting database, which may accelerate research in herbal genomics is Global Pharmacopoeia Genome Database (GPGD), which integrates almost 35 000 records for over 900 herb species from eight global pharmacopoeias [82]. All collected datasets were uniformly formatted and organized in species specific manner. The construction of a model herbs platform seems to be a convenient and useful tool for future research in herbal medicine. As a result, it sets off a storm of new approaches for the efficient identification of medicinal plants through genomic studies.

### Epigenomics

Epigenomics is regarded as a study of heritable changes in gene expression that are not caused by alterations in DNA sequence. In the realm of plant genomes, epigenetic studies, especially referring to DNA methylation, histone modification and chromatin remodeling, offer unprecedented opportunities to comprehend the mechanisms and functions of regulatory pathways [94]. By observing modifications in DNA and chromatin, it becomes possible to determine the active regulatory sequences in plant tissues and at specific stages of plant development. The ability to detect specific epigenetic mechanisms at a genome-wide level can ease the metabolic engineering of alkaloid biosynthesis, particularly in terms of pathway elucidation. Nonetheless, the analysis of epigenetic changes in plants is challenging because it requires the isolation of extremely pure samples of individual cell populations. Consequently, our understanding of epigenetic regulation in plant-specialized metabolism remains relatively unknown [50].

Several studies have reported that among the Papaveraceae family, epigenetic marks have been noticed to take a leading role in the regulation of gene expression and phenotype within the Papaveraceae family. An analysis of DNA methylation profiling in opium poppies, focusing on CCGG loci, revealed that variations in epigenetic modifications among *Papaver* species had minimal impact on the alkaloid profile. However, the authors suggested that demethylation patterns observed in opium poppy species, known for their high levels of BIAs, could be linked to the expression of genes responsible for both defense mechanisms and alkaloid synthesis. Interestingly, distinct organ-specific differences in epigenetic changes were observed, with the capsule or shoot samples showing notable divergence from leaves [95]. In another study, both natural and cultivated populations of *Corydalis yanhusuo* were examined to determine epigenetic variations. The results show higher epigenetic differentiation but lower genetic differentiation in cultivated populations, potentially associated with the different modes of reproduction between natural and cultivated populations of *C. yanhusuo*. Furthermore, alkaloid content varied across cultivated populations and correlated with climatic fluctuations. These results suggest that epigenetic variation could play a significant role in *C. yanhusuo* breeding and contribute to different responses to environmental conditions [96].

Therefore, the discovery of epigenetic patterns will significantly help to understand the regulation of medicinal plant compounds and their potential applications.

### Transcriptomics

Transcriptome refers to the complete set of mRNA molecules, including both protein-coding and noncoding RNAs, expressed from an organism’s genomes within a specific cell, tissue or under defined external conditions [97]. Unlike the genome, the transcriptome provides a snapshot of gene expression at a given time and in a specific cellular or spatial context. The emergence of high-throughput sequencing methods, such as RNA-seq, has revolutionized the study of gene structure, regulation and expression levels in various plant species across different developmental stages and environmental conditions [97]. Advances in sequencing technologies have transitioned gene expression studies from traditional methods like Northern blot or reverse-transcription polymerase chain reaction, which focused on single or a few genes, to more efficient microarray techniques, and now to the analysis of tens of thousands of transcripts using NGS technologies [98]. With the increasing availability of genome information, RNA-seq has become the gold standard for transcriptomics. However, there are still challenges to overcome in RNA sequencing, particularly in the bioinformatic analysis of the generated data. Accurate alignment of reads is a crucial step in calculating precise expression levels of transcripts, which can be challenging for plant species without a fully sequenced genome. It is also worth mentioning that certain plant tissues or organs, such as glandular trichomes, laticifers (especially important in Papaveraceae family), resin ducts and nectaries, have been evolutionarily developed for the production and storage of specialized metabolites. Advancements in single-cell RNA sequencing and laser microdissection techniques are unveiling deeper molecular insights into the gene expression pathways and metabolic specialization within these cells [99]. As an illustration, let’s consider BIAs, such as morphine, which is stored within the laticifers of the opium poppy. Nonetheless, there exists a specialized division of labor among different cell types in the morphine biosynthesis process. While most enzymes responsible for morphine production accumulate within the sieve elements, the corresponding genes are transcribed and translated in adjacent companion cells [100]. Consequently, a mechanism is required to transport these enzymes to the sieve elements, from which they are further transported to laticifers, where the final stages of biosynthesis occur to yield the ultimate products [101]. Such metabolic cell specialization adds another level of complexity to transcriptomic, proteomic and metabolomic studies. In the following chapter, we have focused and summarized recent achievements from transcriptome studies conducted on species belonging to the Papaveraceae family.

Facchini’s laboratory has published two articles investigating transcriptomic data from various plant species that produce BIAs [102, 103]. The authors identified genes involved in BIAs biosynthesis pathways and established resources for discovering homologs or entirely novel enzymes in different plant species. They examined plant species from four families, including 11 species from Papaveraceae, six from Berberidaceae and Ranunculaceae and three from Menispermaceae. In 2012, they described the analysis of gene libraries (~3500 unigenes, which are sets of transcripts from the same transcription locus) acquired from plant cell cultures using the Sanger sequencing method. In 2015, they presented the results of deep sequencing (Roche GS-FLX Titanium and Illumina GA/HiSeq) of cell cultures as well as plant tissues. Based on gene homology, ~850 candidate genes involved in BIAs biosynthesis pathways were preselected. Differential expression analysis of these genes was performed using FPKM (Fragments Per Kilobase of exon model per Million mapped reads) values, enabling the identification of candidate enzymes for potential genetic modifications to enhance or optimize alkaloid synthesis. For example, in *Papaver bracteatum*, two enzymes from the dioxygenase family were identified as candidates expressed at very low levels. This low expression may contribute to a metabolic block, resulting in the accumulation of thebaine and trace amounts of downstream alkaloids, such as codeine and oripavine [103]. Additionally, a taxonomic analysis of 15 gene clusters encoding enzymes involved in alkaloid synthesis allowed the formation of a collection of candidate genes from different plant species. These genes could serve as functional homologs in unicellular systems for alkaloid biosynthesis. Notably, an empirical case study using *Glaucium flavum* N-methyltransferase (NMT) revealed that the proposed phylogenetic approach is an effective tool for function prediction, as it confirmed both predicted and novel enzyme activities [103].

Various factors contribute to the variations in metabolic flow, product yield and tissue-specific accumulation of compounds, which contribute to the increasing complexity of plant metabolism. The flexibility of the transcriptome is influenced by noncoding RNAs, as well as alternative splicing, translation, polyadenylation (AS/AT/APA) and the formation of heterodimers or gene fusions. To capture AS/AT/APA transcriptomic events, third-generation single-molecule real-time sequencing (SMRT) technology, such as Pacific Biosciences, has proven to be a valuable tool. In a study by [47], SMRT sequencing was employed to sequence 21 pooled RNA samples from three different tissues and five different growth phases of the opium poppy. This approach resulted in the generation of 61 856 unigenes, comprising 59 144 protein-coding unigenes and 2712 noncoding unigenes. These sequences were subjected to functional annotation, expanding the transcriptome database for this pharmaceutically important plant species.

The transcriptome of *Dactylicapnos scandens* (D. Don) Hutch, a representative of the Papaveraceae family, was sequenced using the Illumina HiSeq2000 sequencing platform in a study conducted by [104]. The goal of this study was to identify genes encoding enzymes involved in alkaloid biosynthesis in *D. scandens*, which is a TCM herb used for treating inflammation, hypertension, bleeding and pain. The genetic background of this species had limited prior characterization. To facilitate a comprehensive analysis, cDNA libraries were constructed using RNA isolated from the leaves, stems and roots of *D. scandens*. The Illumina sequencing yielded a total of 96 741 unigenes, and among them, 30 190 coding DNA sequences were identified. Leveraging the transcriptome information obtained, the authors proposed integrated biosynthetic pathways for isocorydine, corydine, glaucine and sinomenine, which are IQA responsible for the medicinal effects of *D. scandens* extracts. Similar to the transcriptome analysis conducted on opium poppy, candidate genes involved in the biosynthesis of IQAs were identified, and the transcription factors that control their biosynthesis were described [104].

Transcriptome analyses were also used to identify genes involved in fatty acid biosynthesis in Papaveraceae representatives [105]. The researchers focused on the plant species *C. majus* and investigated the biosynthesis pathways of fatty acids accumulated in elaiosomes, which are nutrient-rich structures attached to seeds. *C. majus* is well-known for its medicinal value and its role in ant-dispersal, known as myrmecochory. For the study, *C. majus* plants at four different developmental stages (flower-bud, flowering, young seed and mature seed) were used. Twelve cDNA libraries were constructed and sequenced, resulting in the generation of 63 064 unigenes. Gene expression level analysis led to the identification of 41 significantly differentially expressed genes (DEGs) involved in lipid metabolism pathways. These DEGs were mostly upregulated in the young seed stage, which corresponds to the patterns of seed cell growth. Detailed analysis of the biosynthesis pathways of linoleic, palmitic and oleic acids revealed candidate genes involved in their production. The research not only sheds light on particular metabolic pathways in *C. majus* but also provides excellent genomic resources for future investigations. For instance, these resources could be utilized to study the convergent evolution of myrmecochorous plants. It is worth mentioning that additional transcriptome resources for *C. majus* are available, including a comparison of leaf and root transcriptomes [106] and an integrated transcriptome and proteome analysis focused on determining the latex composition [107].

Transcriptome studies were also employed to elucidate the resistance mechanisms against downy mildew in *P. somniferum* [108]. This disease, caused by either of the oomycetes *Peronospora meconopsidis* or *Peronospora somniferi*, poses a significant threat to opium poppy cultivation and opium production. In the mentioned research, one group of plants was subjected to treatment with bacterial endophytic strains (SMR1 and SMR2) isolated from *P. somniferum* roots, aiming to enhance resistance. The bacterial endophytes were utilized to induce the production of pathogenesis-related proteins (PRs) or phytoalexins, as well as enhance tolerance against oxidative stress [109, 110], which is often associated with plant diseases. Following RNA isolation and NGS sequencing using the Illumina HiSeq 2000 platform, the comparative transcriptomic analysis revealed differential expression of transcripts encoding defense proteins, proteins involved in signal transduction and those associated with phytohormone production. In conclusion, the authors proposed a salicylic acid-dependent defense pathway, a component of systemic acquired resistance, as the mechanism underlying the increased resistance of opium poppy to the tested pathogens following pretreatment with endophytes.

Transcriptomic studies are often exploited to uncover molecular mechanisms associated with the response of plants to abiotic stress, including drought, which is one of the most detrimental factors affecting crops. *M. cordata*, however, is recognized as a drought-tolerant plant and has even been suggested as a pioneering crop suitable for cultivation in arid conditions. To gain insights into the molecular pathways underlying its tolerance, researchers employed Illumina Hiseq X for transcriptome and degradome sequencing [111]. The authors specifically focused on miRNA analysis, as these single-stranded RNA molecules are known for their involvement in plant stress response. They successfully identified 895 miRNAs, among which 18 showed significant differential expressions between control and drought-treated plants. The target genes of these miRNAs were found to be associated with functional groups, such as glutathione metabolism, plant hormone signal transduction and sulfur metabolism. These findings provide a foundation for comprehending the complex molecular network underlying the drought stress tolerance of *M. cordata*.

Another valuable source of transcriptomic data is the OneKP (or 1KP) project, an international and multidisciplinary initiative that provides information from transcriptome sequencing from 1000 phylogenetically diverse plant species [112]. Within this extensive collection, 33 deposited samples belong to the Papaveraceae family. These samples can be utilized for gene expression analysis or taxonomic studies, providing valuable resources for further research in this field.

### Proteomics

The proteome refers to the complete range of proteins expressed in a cell or tissue. Similar to the transcriptome, it represents a subset of proteins encoded by the genome that are specifically related to developmental conditions or translated in response to external stimuli. Protein expression is influenced not only by the rate of transcription but also by factors, such as mRNA stability and the balance between protein synthesis and degradation. Additionally, protein function can be modulated by posttranslational modifications, including phosphorylation, glycosylation, acetylation and sulfation, occurring at various amino acid residues. These modifications greatly diversify the pool of proteins within cells, but they also make the study of proteins more challenging. While genomic studies are rapidly expanding, large-scale proteomic studies are comparatively less common, primarily due to technological limitations.

An intriguing approach was employed in a study conducted by Onoyovwe [101], using shotgun proteomics to investigate subcellular levels in opium poppy. The researchers utilized a gel-based liquid chromatography–tandem mass spectrometry approach, which enabled the identification of 1518 and 511 distinct proteins in the whole-stem and latex samples, respectively. Through a detailed analysis of the expressed proteins in each subproteome, they discovered the presence of nine enzymes involved in morphine biosynthesis in the whole stem, whereas only four of the final five pathway enzymes were detected in the latex. Notably, among the most abundant proteins in the latex, three of them were the final three enzymes responsible for converting thebaine to morphine. Furthermore, the researchers confirmed the activity of these latex enzymes by observing the conversion of exogenous thebaine in cell-free latex protein extracts. By utilizing immunofluorescent labeling techniques, the authors proposed a mechanism for morphine synthesis in which the biosynthesis of salutaridine (the first tetracyclic promorphinan alkaloid) occurs exclusively in sieve elements, while the conversion of thebaine to morphine is predominantly carried out in adjacent laticifers [101].

Zeng *et al.* [113] conducted comprehensive analyses of the proteome and transcriptome of two Macleaya species, *M. cordata* and *M. microcarpa*. In this study, 10 samples from each species were sequenced, resulting in the annotation of over 60 000 unigenes from the transcriptome data. Through the use of LC–MS/MS, ~4000 peptides were identified, including 1000 nonredundant proteins. The integration of this data with additional information on alkaloid accumulation allowed for the identification of tissues involved in alkaloid biosynthesis at different developmental stages (preflowering stage and the postfruit maturation stage), specifically in roots, leaves and fruits. The findings suggested that roots may serve as the primary organ for alkaloid biosynthesis in *Macleaya spp.* Moreover, the expression of proteins involved in storage and transport was found to be higher in the analyzed tissues, contributing to the accumulation of alkaloids in specific tissues. Nawrot *et al.* [114] employed a tandem mass spectrometry (nanoLC–MS/MS) coupled with label-free protein quantitation (emPAI) approach to investigate *C. majus* and *Corydalis cava*, two representatives of the Papaveraceae family. By analyzing the shoot extract of *C. majus* and the tuber extract of *C. cava*, the researchers were able to identify 1240 and 228 proteins, respectively. Notably, both proteomes exhibited a relatively high abundance of stress-responsive and defense-responsive proteins. Additionally, both extracts revealed the presence of various PRs and low molecular weight inducible antimicrobial peptides, which could potentially interact with alkaloids in the plant’s defense mechanisms.

To unravel the antiviral activity of *C. majus* extracts, Nawrot [24] analyzed latex from five different developmental stages using an LC–MS-based label-free proteome approach. During the analysis of each set of proteins, the authors observed a shift in the expression of proteins involved in various biosynthesis processes, such as protein synthesis, transcription, protein folding and active transport of molecules. This shift was observed from the flowering phase to the fruit ripening and seed maturity phases, where proteins associated with plant defense pathways became more prominent. These findings suggest that *C. majus* extracts may have medicinal uses not only during its flowering period but also during the fruit ripening phase, due to the activation of plant defense pathways.

### Metabolomics

The term ‘metabolome’ was coined in 1998 by Steven Oliver, marking a significant shift in research approaches across various biomedical fields [115, 116]. Metabolomics, as part of the -omics movement, has emerged as an effective tool for studying the chemical composition of plants and the biochemical processes in which plant metabolites participate. Within the realm of herbgenomics, metabolomics plays a crucial role in analyzing the synthesis and regulation of bioactive plant metabolites. By understanding the genetic foundations and control mechanisms of biosynthetic pathways, supported by NGS, it becomes possible to overcome challenges associated with low-level synthesis and produce pharmaceutically important metabolites in larger quantities [117].

The investigation of chemical composition in several members of the Papaveraceae family has been explored using metabolomics approaches and various analytical tools, such as gas chromatography–mass spectrometry (GC–MS), liquid chromatography-mass spectrometry (LC–MS), capillary electrophoresis-mass spectrometry (CE-MS) and nuclear magnetic resonance (NMR) spectroscopy. Notable studies in this area are summarized in Table 2 [41, 62, 118, 119].

**Table 2:** Selected metabolites identified in Papaveraceae plants using metabolomic techniques

Papaveraceae species		Technique used	Identified metabolites	Reference
Common name	Latin name			
Opium poppy	*Papaver somniferum*	HPLC	Alkaloids: morphine, codeine, thebaine, papaverine, noscapine Flavonoids: kaempferol, quercetin	[62, 122]
–	*Corydalis yanhusuo*	2D, LC, HPLC-MS/MS	Alkaloids: dehydrocorydaline, tetrahydro proto papaverine, coptisine, columbamine, protopine, palmatine, canadine	[123]
Red poppy/corn poppy	*Papaver rhoeas*	LC-QTOF-MS/MS	Alkaloids: protoberberine, aporphine, benzylisoquinoline, protopine, stylopine	[118]
California poppy, golden poppy	*Eschscholzia californica*	HPLC	Alkaloids: sanguinarine, chelirubine, macarpine, chelerythrine, chelilutine	[124]
Fumewort	*Corydalis solida*	LC-ESI-MS/MS	Alkaloids: berberine, coptisine, allocryptopine, chelidonine, protopine derivative, chelidonine derivative, chelerythrine, tetrahydroberberine, tetrahydrocortisone, sanguinarine, protopine Flavonoids: quercetin, rutin Other compounds: chlorogenic acid *p*-coumaric acid, *trans*-caffeic acid	[119]
Yellow corydalis	*Pseudofumaria lutea*	LC-ESI-MS/MS	Alkaloids: protopine derivative, coptisine, berberine, chelidonine, chelerythrine, tetrahydroberberine, tetrahydrocoptisine, sanguinarine Flavonoids: quercetin, rutin Other compounds: *trans*-caffeic acid, chlorogenic acid, *p*-coumaric acid	[119]
Greater celandine	*Chelidonium majus*	HPLC-DAD, LC–MS/MS	Alkaloids: chelerythrine, sanguinarine, coptisine	[125]
		HPLC-DAD, MRM	quercetin, phenolic acids	[126]
		LC/MS-TOF	Alkaloids: protopine, coptisine, berberine, chelidonine, chelerythrine, sanguinarine, allocryptopine, norchelidonine, canadine	[127]

Metabolomic studies focusing on morphine derived from *P. somniferum* have received significant attention due to its analgesic properties and the negative consequences associated with its abuse. These studies have led to the identification of multiple genes and enzymes involved in morphine biosynthesis. Genetic manipulation of these genes holds promise for increasing the quantity and purity of morphine in large-scale production. Overall, metabolomic approaches provide valuable insights into the complex biosynthetic pathways of morphine and related alkaloids in the opium poppy. Additionally, the effects of genetic engineering on these pathways have been explored using metabolomic techniques. Notably, metabolomic approaches have been used to enhance morphine production in genetically engineered plants [27, 62]. It is worth noting that low morphine opium poppy genotypes have also been developed, which may have applications in the food industry [120].

Metabolomics studies have been conducted on the California poppy (*E. californica*) to investigate the presence and levels of various metabolites and their potential health benefits. Notably, *E. californica* has become a promising model organism for studying the regulation of morphogenesis and secondary metabolism, thanks to the development of effective protocols for cell and tissue culture. Liscombe and coworkers created a platform to identify new biosynthetic genes by analyzing transcript and alkaloid profiles in cell cultures of *E. californica* and *P. bracteatum* [121].

To examine the BIA metabolic networks, a combination of ESI-FTICR-MS and ESI-MS/MS analyses was employed. These analyses revealed the diverse nature of BIA metabolic networks, consisting of both known and predicted biosynthetic branches. By integrating transcriptomic and metabolomic profiles, researchers were able to map the chemical and biochemical constituents of the alkaloid pathways. This comprehensive approach facilitated the identification and functional characterization of several NMTs involved in the biosynthesis of alkaloids.

Metabolomic research has also been conducted on *C. majus*. In a study conducted by Warowicka *et al.*, a combination of high-performance liquid chromatography-mass spectrometry (HPLC/ESI-MS) and NMR was employed to determine the composition of protoberberine-rich fraction (BBR) obtained from celandine [42]. The BBR was then used to investigate its effectiveness in the treatment of endometriosis in rats. The study identified several alkaloids in the BBR, including berberine, coptisine, chelidonine, sanguinarine and stylopine.

Further experiments conducted by the authors showed that the supplementation of BBR led to the regression of endometriosis loci and the complete removal of all visible ectopic endometriosis lesions in the rats. This -omics-based approach suggested that BBR supplementation could effectively prevent the recurrence of endometriosis in the rat model [42].

In a study conducted by [119], the phytochemical components of two Papaveraceae species, *Corydalis solida* and *Pseudofumaria lutea*, were analyzed and compared. Liquid chromatography-electrospray ionization-tandem mass spectrometry (LC-ESI-MS/MS) was employed, and a total of 21 compounds were identified. The identified compounds included various alkaloids. such as derivatives of protoberberine (e.g. coptisine and berberine), derivatives of protopine (e.g. protopine and allocryptopine) and derivatives of phenanthridine (e.g. sanguinarine, chelerythrine and chelidonine), as well as two hydroxycinnamic acids (*p*-coumaric acid and *trans*-caffeic acid), three carboxylic acids (*trans*-aconitic acid, malic acid and quinic acid), one phenolic aldehyde, one chlorogenic acid and two flavonoids (rutin and quercetin) [119].

The content of individual compounds differed significantly between the two species. *C. solida* exhibited a broader variety of compounds present in lower quantities, while *P. lutea* extracts contained fewer compounds but in larger amounts. Protopine was found to be one of the most abundant constituents in both *C. solida* (440–1125 μg/g d.w.) and *P. lutea* (1036–1934 μg/g d.w.). In the aerial parts of *P. lutea*, substantial amounts of coptisine (1526 μg/g) and quercetin (3247 μg/g) were also detected.

Furthermore, the antimicrobial potential of extracts from the aerial and underground parts of both species was evaluated against *Staphylococcus aureus*, *Pseudomonas aeruginosa* and *Candida albicans*. Among all three pathogens, the extract from *P. lutea* showed the highest effectiveness, with a MIC of 0.39 mg/L [119].

The integration of metabolomics with transcriptomics and proteomics has proven to be a powerful approach in accelerating research on metabolites and the pathways they are involved in within the Papaveraceae family. These herbgenomics tools enable the identification of genes responsible for the biosynthesis pathways of plant metabolites.

### Herbgenomics as an approach in enhanced Papaveraceae biopharmaceutical production

**Figure 2:**
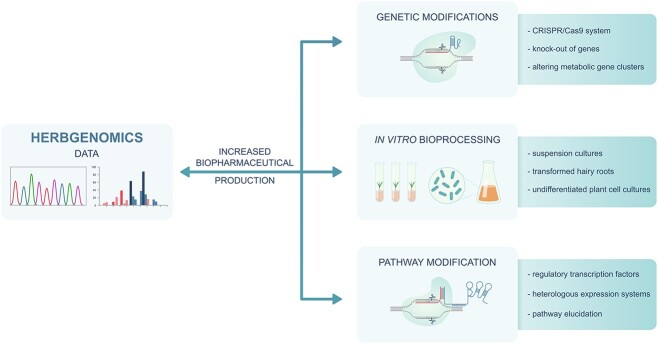
Potential applications of herbgenomics data in order to enhance production of biopharmaceuticals isolated from Papaveraceae herbs.

Undoubtedly, the utilization of herbgenomics methodology and the integration of data obtained from sequencing, expression regulation and protein interactions can greatly accelerate the discovery of biochemical pathways and enzymes involved in BIAs metabolism, as well as streamline of BIAs. This approach can also facilitate the large-scale production of previously known molecules. Additionally, the use of an -omics approach enables the investigation of microRNAs, cryptic biosynthetic gene clusters or data chromatin organization, all of which have the potential to enhance the production of phytochemicals [128]. The augmentation of Papaveraceae-specific compounds through herbgenomics is predicated on investigation of plant molecular genetic basis and manipulating their genetic background or physiology to regulate the biosynthesis of biopharmaceuticals [117]. This chapter discusses several principal approaches, including genetic modifications, metabolic engineering, bioprocessing, the utilization of heterologous expression systems and pathway modifications with the focus on their elucidation (Figure 2).

*In vitro*, bioprocessing methods, such as suspension culture, transformed hairy roots and pathway elucidation, have proven effective in achieving high yields of desired metabolites, including berberines, shikonins and cinnamic acid derivatives. These metabolites exhibit robust growth in suspension cultures [129, 130]. However, the enhanced production of morphinan alkaloids, a hallmark of *P. somniferum*, remains a challenge as they are expressed only in trace amounts [131]. Shoot cultures have shown promise in achieving higher quantities of these alkaloids. The use of elicitors, such as methyl jasmonate, yeast extract and salicylic acid, has been successful in augmenting the accumulation of dihydrosanguinarine and sanguinarine in *E. californica* [132]. Additionally, fungal elicitors have been utilized to accumulate benzophenanthridine alkaloids (such as sanguinarine and/or macarpine) in Papaveraceae species. The discovery of cytochrome P450-dependent enzymes like monooxygenase dihydrochelirubine-12-hydroxylase and SAM-dependent 12-hydroxydihydrochelirubine-12-O-methyltransferase in *S. canadensis* and *E. californica* has provided additional opportunities for enhancing alkaloid production through elicitation by *Thalictrum bulgaricum* yeasts. Moreover, in opium poppy cell cultures, elicitor treatments and transient expression of members of the tyrosine decarboxylase (TYDC) gene family, including CYP80B1, have been explored [133].

The advent of new molecular tools and postgenomic -omics approaches has ushered in a new era of research. In contrast to the pre-genomic era, which relied mainly on biochemical approaches, modern techniques, such as positional cloning, tagging, expression libraries and homology-based cloning, have been developed to complement these traditional methods. They have enabled the elucidation of molecular mechanisms through metabolic bioengineering by providing data on genes encoding regulatory factors and enzymes that catalyze the biosynthesis of secondary metabolites. Another approach that can be used to obtain morphine alkaloids is based on elicitation of alkaloids by using undifferentiated plant cell lines, which have the capacity to produce the same alkaloids as mature plants on an industrial scale [134].

The use of -omics techniques, coupled with the decreasing cost of DNA sequencing, have aided in detection of tissues, such as leaf trichomes, which are major sites for the synthesis of various secondary metabolites [135]. Analysis of plant genomes and the examination of specific alkaloids may lead to the creation of novel analogs by mutasynthesis by functional group substitution. The determination of enzymatic steps, through tracing isotopically labeled metabolites along with reverse genetics to associate metabolites with corresponding genes, enzymatic steps in biosynthetic pathways can be determined, aiding in the identification of biosynthetic transformations [134]. Alterations of biosynthetic pathways, due to its complexity can lead to increase in desired product synthesis and unexpected amplification of undesired by-products. In the Papaveraceae family, BIAs derived from tyrosine are prevalent. The biosynthetic origin of diverse BIAs can be traced back to the Pictet-Spengler condensation of 4-hydroxyphenylacetaldehyde dopamine, which is derived from tyrosine. The intermediate molecule, (*S*)-norcoclaurine, undergoes further modifications, leading to the formation of various structural frameworks. Through the condensation of dopamine, followed by a series of methylations and hydroxylations, the final intermediate molecule, (*S*)-reticuline, is produced, serving as a precursor for the synthesis of various alkaloids [134, 136]. The integration of information from metabolomics, proteomics and transcriptomics enables the engineering of metabolic pathways with more predictable outcomes. Overexpression of COR1 enzyme, which encodes codeinone reductase involved in morphine biosynthesis, led to moderate increase in synthesis of both morphine and codeine in opium poppy, while unexpectedly increased production of thebaine [137]. Overexpression of the gene *CYP80B3*, encoding the (*S*)-*N*-methylcoclaurine 3′-hydroxylase isozyme 1, caused a significant 450% increase in the total morphine-related alkaloids [138]. Gene silencing using a chimeric small hairpin RNA construct targeting a multigene family encoding codeinone reductase resulted in the accumulation of the precursor alkaloid (*S*)-reticuline and enhanced production of morphine, codeine, oripavine and thebaine [139]. In *E. californica*, RNAi-mediated suppression of berberine bridge-forming enzymes led to the silencing of these enzymes and the accumulation of reticuline, resulting in reduced levels of sanguinarine and increased levels of laudanine [140].

To promote the progress of herbgenomics and bridge the gap between existing knowledge of microbial biology and its application in enhancing biopharmaceutical production, the introduction of heterologous expression systems into metabolic engineering can be employed. Using microbial systems, such as *Escherichia coli* and the yeast *Saccharomyces cerevisiae*, offers significant advantages due to their well-known biochemistry, availability of genome sequences, gene clustering and lower complexity compared to plants. Interestingly, endophytes, which are microbes naturally found in various plant tissues, can produce the same phytochemicals as their host plants. This further facilitates biosynthetic production, as endophytes often possess highly similar homologous genes to those in the host plant [128]. *E. coli* and yeast *S. cerevisiae* have been used to produce plant alkaloids due to their lower degree of complexity and smaller genome size. These species have been employed to modify BIA pathways [134]. For example, introducing plasmids into the yeast genome that express genes encoding enzymes, such as 6-OMT, CNMT and 4′-OMT involved in the formation of the (*S*)-reticuline precursor of opium BIA ramification has led to the heterologous expression of artificial plant pathways with maintained high catalytic activities [141, 142]. To target the production of (*S*)-norcoclaurine, which is catalyzed by an NCS enzyme and serves as a precursor to all BIAs, Bourgeois *et al.* codon-optimized the PsNCS1 and PsNCS3 orthologs from *P. somniferum* for overexpression in yeast using a synthetic landing pad system. This led to all *S. cerevisiae* mutants secreting (*S*)-norcoclaurine [143, 144]. A significant advantage of yeast over *E. coli* is the ability to support the functionality of cytochrome P450 enzymes, which require posttranscriptional modifications and are vital in morphine metabolism. Overexpression in *E. coli* was also shown to increase thebaine levels by 300-fold compared to yeasts, primarily due to increased expression of SalSyn, Sa1AT and SalR, which are genes involved in the final steps of salutaridine-related enzyme genes in morphine biosynthesis. The use of (*R*,*S*)-tetrahydropapaveroline as an intermediate allowed for the establishment of new molecular pathways for the production of (*R*)-reticuline, a precursor to BIAs, including thebaine [145].

The ectopic heterologous expression of *CjWRKY1* using *E. californica* cells has shown promising results in increasing the transcription of multiple BIA genes encoding biosynthetic enzymes, resulting in an increased concentration of various alkaloids in the culture medium, including chelerythrine, chelirubine, protopine, allocryptopine, sanguinarine and hydroxychelerythrine [146]. However, a limitation of using microbes for alkaloid production is the decline in secondary metabolite production after a few generations, which may be related to the lack of horizontal transfer of genetic material between the host plant and endophytic microbes [128].

Additionally, the toxicity of the produced alkaloids can hinder the growth of microorganisms, thereby impacting the overexpression of secondary metabolites. Transcriptomic approaches have been employed to analyze the metabolite toxicity of alkaloids produced, aiming to understand and address these issues [147].

A significant milestone in the field of metabolic engineering of Papaveraceae was achieved with the introduction of Clustered Regularly-Interspaced Short Palindromic Repeats (CRISPR/Cas9) technology, which was awarded the Nobel Prize in Chemistry. Compared to other genome editing methods, such as zinc finger nucleases or TALEN, the CRISPR/Cas9 system is recommended for editing plant genomes due to its simplicity, efficiency, reduced cost and the ability to combine target genes to accelerate the modification process [148–150]. The integration of herbgenomics data has facilitated the identification of 40 000 expressed enzyme tags, enabling the isolation of genes involved in alkaloid pathways [62].

Using the type II CRISPR/SpCas9 system, [151] successfully knocked out the 4′OMT2 gene in the opium poppy, which is responsible for regulating BIAs. This knockout resulted in a significant decrease in (*S*)-reticuline and laudanosine, adversely affecting morphine biosynthesis. Interestingly, besides the reduction in morphine and thebaine biosynthesis, a novel alkaloid was identified in the obtained mutants [152]. Agroinoculation with sgRNA and Cas9 using the TRV vector inside plant tissues allowed for the manipulation of BIA production flux to engineer metabolic biosynthesis [151]. In another study, several BIA biosynthesis-related genes, including *EcG3OMT* and *ECG11OMT*, were significantly increased, while *EcCYP2* showed a moderate increase. Notably, preferential transcriptional modulation of *EcCYP1* was observed, along with the involvement of EcCZP719A3 and EcG110MT transcription factors in the rate-limiting step of BIA biosynthesis [153]. Additionally, overexpression of *CjWRKY1* genes facilitated BIA biosynthesis in *E. californica* [146].

In the tanshinone biosynthesis pathway, knocking out SmCPS1 resulted in homozygous mutants with no major tanshinones, such as tanshinone I, tanshinone IIA and cryptotanshinone. Chimeric mutants also exhibited reduced levels of tanshinones [154, 155]. Genomic data analysis allows for screening the genome for candidate gene clusters involved in the biosynthesis of plant natural products. Through analysis of high noscapine-producing *P. somniferum* genomic data, a cluster of 10 genes spanning 221 kb was discovered, further characterizing crucial genes involved in noscapine biosynthesis [63]. These studies also revealed the catalysis order in the noscapine biopathway, providing a novel biosynthetic pathway for this alkaloid [63].

By combining data from sequencing, protein interactions and other -omics techniques, a better understanding of genes involved in biochemical pathways has been achieved, providing a long-term perspective on the discovery of plant-based drugs. Pharmaceuticals of natural origin continue to play a significant role in new drug investigations, with 33.5% of drugs used in cancer treatment from 1981 to 2020 being derived from natural sources. This percentage increases to 64.9% when considering their synthetic modifications [156].

## CONCLUSION

The purpose of this paper is to present a new approach called herbgenomics, leveraging recent technological developments in life science. This approach holds the potential to make significant contributions to the field of medicinal plants and may facilitate the discovery of new phytochemicals within the Papaveraceae family. In the most probable scenario, some herbs from this family could serve as targets for the development of future plant-based therapeutic agents. Consequently, it is imperative to conduct further research from an -omics perspective, focusing on the investigation of plant metabolites, their biological properties, as well as the molecular mechanisms underlying their biosynthesis and regulation at various levels.

In addition to the discovery of new biopharmaceuticals, herbgenomics enables effective metabolic engineering strategies to achieve specific molecule production in plants. The use of transcription factors able to cause simultaneous changes in multiple metabolic points, mathematical models of plant metabolism and system biology analyses will allow for effective alkaloid production [157]. Understanding the regulatory mechanisms, such as DNA methylation, microRNA expression and the role of transport proteins, can help to tinker biosynthetic pathways [128]. It is important to note the differences between pathways and plant species when considering the regulation of biosynthetic pathways by transcription factors. Furthermore, accurate characterization of promoter regions and their sequences will be necessary for future research [146]. Developing genetically engineered medicinal plant varieties with desired traits is the first step towards ensuring high-quality herb cultivation. The herbgenomics approach not only enables accurate identification of metabolic pathways to create superior varieties but also enhances cultivation management and cultural practices under proper environmental conditions [117]. Knowledge of genome sequences and associated data, including metabolite profiles, will facilitate the discovery of biosynthetic pathways for beneficial plant metabolites and potentially reveal new bioactive compounds with significance in pharmaceutical science.

Although herbgenomics provides numerous datasets, the predictability of genetic transformations is limited due to cellular complexities. Despite significant progress in plant molecular biology and the increased availability of -omics data, the application of such methods to medicinal plants remains scarce. In the case of Papaveraceae, the aim is currently on producing a high quantity of medically important biomolecules, with less emphasis on improving crop yields [128]. Standardized protocols for stable genetic transformation and subsequent regeneration of mature Papaveraceae plants are lacking. Recently, [157] established an efficient method for the genetic transformation of *E. californica*, which involved protoplast isolation and somatic embryo induction. This method enables gene function analysis and the use of stable transgenic lines for reproducible results through CRISPR/Cas9 technology [158]. However, each -omics technology has its advantages and disadvantages. Even the CRISPR/Cas9 method has limitations, such as the low-cargo capacity of plant viral vectors [151].

Fortunately, with the decreasing cost of genome sequencing and assembly, the number of sequenced genomes of important medicinal plants is expected to increase. The combination of more sequenced medicinal plant genomes, metabolomics, and other -omics approaches will facilitate faster and more cost-effective screening and biosynthesis of plant secondary metabolites, benefiting human and animal well-being. We eagerly anticipate the translation of these scientific discoveries into clinical applications.

**Key Points**

- Papaveraceae is a plant family well known for medicinal properties of its representatives, attributed to diverse bioactive compounds found in their latex.
- Integration of genomic data with other -omics studies has emerged of herbgenomics, which led to understanding the genetic basis of medicinal properties, including essential genes and secondary metabolite biosynthesis pathways.
- Advances in high-throughput technologies and genetic manipulation techniques like CRISPR/Cas9 enable systematic studies, targeted breeding, gene banks and synthetic biology approaches for medicinal plants.

## Data Availability

Not applicable.

## Notes

**Natalia Kielich** is a PhD student at Adam Mickiewicz University in Poznań and her current research interests are concentrated on genetic manipulation in medicinal plants.

**Oliwia Mazur** is a PhD student at Adam Mickiewicz University in Poznań focused on unraveling the biopharmaceutical potential of *Chelidonium majus* latex compounds.

**Oskar Musidlak** after finishing a PhD in research related to therapeutic effect of *C. majus* latex against HPV, is working in the Department of Molecular Virology, Adam Mickiewicz University in Poznań. He participates in several projects in the fields of bacteriophages and plant latex proteins.

**Joanna Gracz-Bernaciak** is a postdoc in the Department of Molecular Virology, Adam Mickiewicz University in Poznań. In particular, Dr Gracz-Bernaciak is interested in plants’ molecular response to biotic as well as abiotic stress conditions.

**Robert Nawrot** is an associate professor, Head of the Molecular Virology Department at the Faculty of Biology, Adam Mickiewicz University in Poznań since 2020. His research is focused on plant defense-response against viral and microbial infection using a model of medicinal plant *C. majus* (Papaveraceae) and its latex. Recently, he participated in the Kosciuszko Foundation Exchange Program to the US, UNC Chapel Hill, NC, USA.
